# Oncolytic Reovirus and Immune Checkpoint Inhibition as a Novel Immunotherapeutic Strategy for Breast Cancer

**DOI:** 10.3390/cancers10060205

**Published:** 2018-06-15

**Authors:** Ahmed A. Mostafa, Daniel E. Meyers, Chandini M. Thirukkumaran, Peter J. Liu, Kathy Gratton, Jason Spurrell, Qiao Shi, Satbir Thakur, Don G. Morris

**Affiliations:** 1 Department of Pathology and Laboratory Medicine, University of Calgary, 2500 University Drive NW, Calgary, AB T2N 1N4, Canada; ahmed.mostafa@cls.ab.ca; 2 Histocompatibility and Immunogenetics, Calgary Lab Services, 3535 Research Road NW, Calgary, AB T2L 2K8, Canada; 3 Department of Oncology, University of Calgary, 1331 29 Street NW, Calgary, AB T2N 4N2, Canada; daniel.meyers@ucalgary.ca (D.E.M.); cmthiruk@ucalgary.ca (C.M.T.); kgratton@ucalgary.ca (K.G.); scienceofkungfu@gmail.com (J.S.); sthakur@ucalgary.ca (S.T.); 4 Tom Baker Cancer Centre, 1331 29 Street NW, Calgary, AB T2N 4N2, Canada; qiao.shi@albertahealthservices.ca; 5 Faculty of Medicine, University of Toronto, King’s College Circle, Toronto, ON M5S 1A8, Canada; peterjr.liu@mail.utoronto.ca

**Keywords:** oncolytic viruses, reovirus, immune checkpoint inhibition, PD-1, breast cancer, immunotherapy

## Abstract

As the current efficacy of oncolytic viruses (OVs) as monotherapy is limited, exploration of OVs as part of a broader immunotherapeutic treatment strategy for cancer is necessary. Here, we investigated the ability for immune checkpoint blockade to enhance the efficacy of oncolytic reovirus (RV) for the treatment of breast cancer (BrCa). In vitro, oncolysis and cytokine production were assessed in human and murine BrCa cell lines following RV exposure. Furthermore, RV-induced upregulation of tumor cell PD-L1 was evaluated. In vivo, the immunocompetent, syngeneic EMT6 murine model of BrCa was employed to determine therapeutic and tumor-specific immune responses following treatment with RV, anti-PD-1 antibodies or in combination. RV-mediated oncolysis and cytokine production were observed following BrCa cell infection and RV upregulated tumor cell expression of PD-L1. In vivo, RV monotherapy significantly reduced disease burden and enhanced survival in treated mice, and was further enhanced by PD-1 blockade. RV therapy increased the number of intratumoral regulatory T cells, which was reversed by the addition of PD-1 blockade. Finally, dual treatment led to the generation of a systemic adaptive anti-tumor immune response evidenced by an increase in tumor-specific IFN-γ producing CD8^+^ T cells, and immunity from tumor re-challenge. The combination of PD-1 blockade and RV appears to be an efficacious immunotherapeutic strategy for the treatment of BrCa, and warrants further investigation in early-phase clinical trials.

## 1. Introduction

Despite recent advances in the diagnosis and treatment of breast cancer (BrCa) [1], the burden of this disease remains significant. During 2018, it is estimated that approximately 266,000 women will receive a BrCa diagnosis, accounting for almost 41,000 fatalities in the USA alone [2]. Although the five-year relative survival of women diagnosed with localized breast cancer is almost 99%, regional and distant-stage disease have five-year survival rates of 84% and 27%, respectively [2]. As such, novel non-surgical treatment options for these patients are needed.

Despite the original consideration of BrCa as immunologically ‘silent’, it is now understood that the host immune system is integral to the efficacy of BrCa therapy, especially in the triple-negative subtype [3,4,5]. Thus, the ability to elicit a potent anti-tumor immune response serves as a logical treatment strategy for this disease. Oncolytic viruses (OV) are a novel group of cancer therapeutics that have the ability to induce such an immune response, and have demonstrated minimal human toxicity to date.

Reovirus (RV), a ubiquitous double-stranded RNA virus, has shown oncolytic potential against over 80% of human cancer cell lines tested [6], and is not known to be associated with any human disease—making it a promising OV candidate. Its tumor tropism is multi-dimensional, as it has been shown that Ras pathway mutations [7,8], as well as overexpression of Junctional Adhesion Molecule-A (JAM-A) [9], permit effective RV cell entry and oncolysis. Further, non-malignant cells lines have been shown to be non-permissive to RV-mediated oncolysis in vitro [10,11]. Additionally, RV has been shown by both our group and others to have extensive preclinical and clinical efficacy against a number of cancer histologies [11,12,13,14,15,16,17], underscored by the establishment of a potent anti-tumor immune response. Furthermore, a randomized phase II clinical trial of RV + paclitaxel in metastatic BrCa has been completed by the Canadian Clinical Trial Group (CCTG). Final data analysis from this study indicate a significant benefit to median overall survival in the treatment group that received RV + paclitaxel (17.4 months) compared to paclitaxel alone (10.4 months) [18]. As such, further exploration of RV as part of a strategy for BrCa is warranted.

Therapeutic targeting of immune checkpoint proteins (i.e., PD-1/PD-L1 axis proteins) has had clinical success across a number of solid tumor types [19,20,21,22]. Recently, it has been discovered that PD-L1 is overexpressed in up to 20% of patients with BrCa, and this may contribute to a worse prognosis [23,24,25]. Although clinical trials are underway to investigate the efficacy of targeting the PD-1/PD-L1 axis in BrCa, the combination of immune checkpoint inhibition and other immunotherapeutic strategies, such as RV, to produce a more robust anti-tumor immune response is of interest. This treatment strategy has been successful pre-clinically in a number of other tumor types [26,27,28,29], likely relating to an OV-mediated CD8^+^ T-cell anti-tumor response and a concomitant up-regulation of tumor PD-L1 expression [30,31].

We seek herein to investigate the utilization of both RV and PD-1 blockade as an effective synergistic immunotherapeutic strategy for localized BrCa in an immunocompetent syngeneic murine model. Our work highlights the potential for this combinatorial treatment approach, and supports its investigation in humans as part of a phase I/II clinical trial.

## 2. Results

### 2.1. Reovirus Infection of Human and Murine Breast Cancer Cell Lines Result in Oncolysis, Cytokine Production and Immune Cell Migration

**Figure 1:**
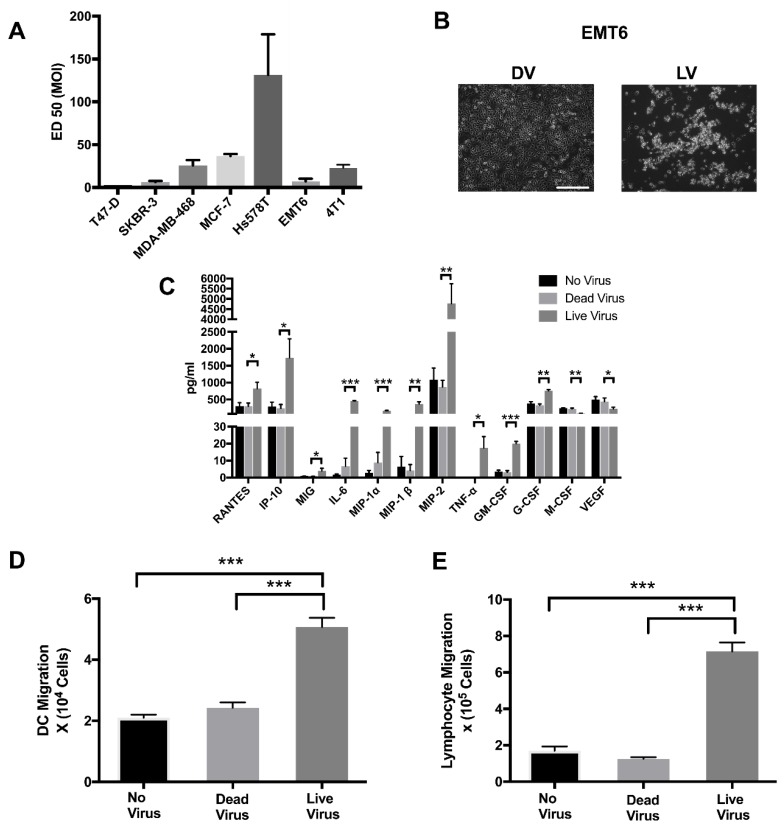
Reovirus has both direct oncolytic effects and induces an inflammatory immune response in breast cancer cells. (**A**) ED_50_ of established human and murine breast cancer cell lines infected with serial dilutions of reovirus (RV) multiplicity of infection (MOI) and incubated for 48 h. Cytotoxicity was detected by measuring mitochondrial NADPH dehydrogenase using a (2-(4-iodophenyl)-3-(4-nitrophenyl)-5-(2,4-disulfophenyl)-2H-tetrazolium, monosodium salt (WST) assay. *N* = 3 per group. (**B**) EMT6 cells infected with ED_50_ (7.37 MOI) of UV-irradiated dead reovirus (DV) or live reovirus (LV) for 48 h, taken with a Zeiss Axiovert 200M microscope at 10× zoom. Scale bar = 50 μm. (**C**) EMT6 cells +/− ED 50 (7.37 MOI) of DV or LV and incubated for 24 h. Chemokine and cytokine levels in supernatants from EMT6 cells were determined by luminex analysis. *N* = 3 per group. (**D**) Dendritic cell or (**E**) Lymphocyte migration in response to cytokine secretion from EMT6 infected or not by RV using of a Transwell^®^ migration assay. *N* = 4 per group. *** *p* ≤ 0.001, ** *p* ≤ 0.01 and * *p* ≤ 0.05 by one-way ANOVA. Error bars = standard error of the mean (SEM) of three independent experiments.

To determine the oncolytic activity of reovirus (RV) against breast cancer, a panel of human and murine breast cancer cell lines were tested for RV mediated oncolysis. T-47D, SKBR-3, MDA-MB-468, MCF-7, Hs 578T, EMT6 and 4T1 were infected with RV at multiplicity of infection (MOI) between 0.3 and 300 and the effective dose for 50% cytotoxicity (ED50) values were calculated using a WST-1 cytotoxicity assay. As shown in Figure 1A, T-47D and SKBR-3 were highly sensitive to RV, while Hs 578T was the least sensitive to RV. Both MDA-MB-468 and MCF-7 experienced a moderate sensitivity to RV. Similar to the human breast cancer cell lines, infection of EMT6 and 4T1 cells with RV resulted in significant oncolysis as detected by WST-1 (Figure 1A). An example of the cytopathic effect of RV on BrCa was demonstrated by light microscopy of EMT6 cells post-RV infection, highlighted by cell blebbing, cell rounding, vacuolization and detachment (Figure 1B).

Not only is RV capable of inducing a direct oncolytic effect on a variety of cancer cells including breast, prostate and renal cell carcinoma [11,13,32], but it can also initiate an immune response through induction of a variety of pro-inflammatory cytokines. To assess RV efficacy on induction of pro-inflammatory cytokines in murine breast cancer cells in preparation for an in vivo study, EMT6 cells were infected with RV for 24 h. Supernatants were collected and chemokine secretions were examined by a luminex assay. RV significantly induced the production of pro-inflammatory chemokines, RANTES, IP-10, MIG, IL-6, MIP-1-α, MIP-1-β, MIP-2, TNF-α and GM-CSF compared to uninfected cells (Figure 1C). Interestingly, RV significantly reduced the inflammatory cytokine M-CSF and the angiogenic cytokine VEGF. Furthermore, using the Transwell™ migration assay we were able to demonstrate that supernatant collected from EMT6 cells infected with live RV (LV) increased the chemotactic activity of dendritic cells (DCs) (Figure 1D) and lymphocytes (Figure 1E) compared to supernatant from EMT6 infected with UV-Inactivated RV (DV) or no virus controls.

Given EMT6’s higher susceptibility to RV, potential to induce potent cytokine production, mediate leukocyte migration, and known immunogenicity [33], it was chosen for the in vivo model.

### 2.2. Reovirus Induces Expression of PD-L1 on the Surface of Human and Murine Breast Cancer Cell Lines Independent of the Presence of Virus

As productive RV infection induces the cellular production of interferon, which is linked to the modulation of PD-1/PD-L1 axis proteins [34], we sought to assess the effect of RV on the expression of PD-L1 in human and murine BrCa cell lines.

**Figure 2:**
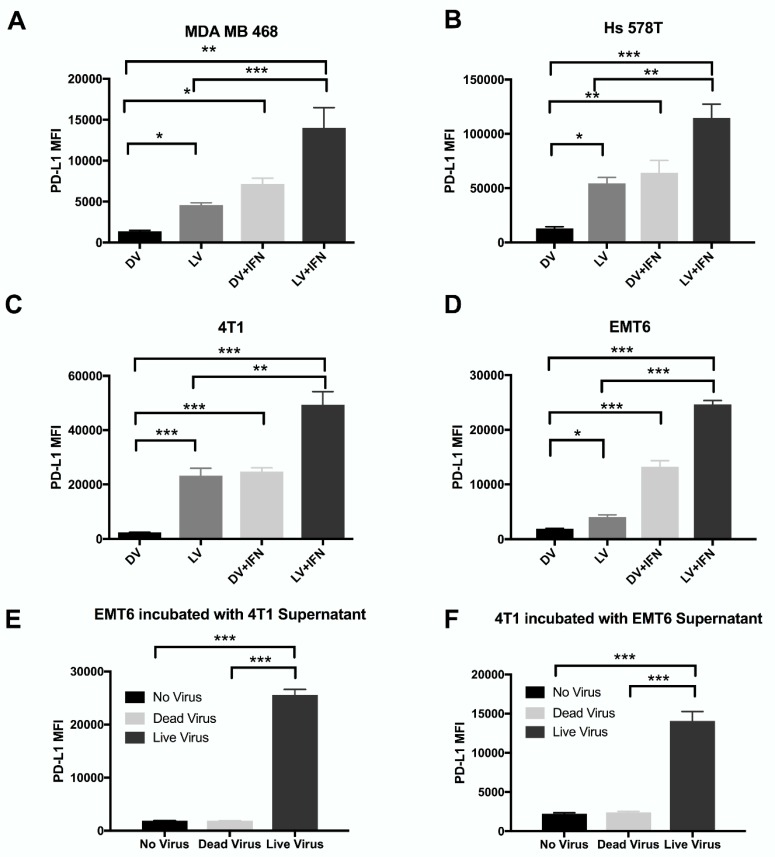
Reovirus modulates PD-L1 expression on breast cancer cell lines. Human (**A**) MDA-MB-468 (**B**) Hs 578T and murine (**C**) 4T1 (**D**) EMT6 breast cancer cell lines were either treated with ED_50_ RV +/− IFN-γ or DV +/− IFN-γ. Expression of surface PD-L1 was analyzed via surface flow cytometry. *N* = 3 per group.(**E**) EMT6 or (**F**) 4T1 cells were incubated with UV-inactivated supernatant from 4T1 or EMT6 cells, respectively, previously treated with RV or DV for 24 h. PDL-1 expression was analyzed by surface flow cytometry. *N* = 3 per group. *** *p* ≤ 0.001, ** *p* ≤ 0.01 and * *p* ≤ 0.05 by one-way ANOVA. Error bars = SEM of three independent experiments.

In all cell lines tested, RV significantly increased PD-L1 levels compared to DV control. (Figure 2A–D) This increase in PD-L1 expression was significantly augmented by the addition of IFN-γ. Additionally, IFN-γ induced PD-L1 expression similarly to the DV + IFN-γ treatment group (Data not shown). These data suggest that productive RV infection can induce PD-L1 expression on both human and murine BrCa cell lines. Furthermore, these data provide rationale for combining RV with PD-1/PD-L1 blockade to augment the anti-tumor immune response secondary to RV-mediated oncolysis.

Although Figure 2 demonstrates RV’s ability to upregulate PD-L1 expression in both human and murine BrCa cell lines, it was unknown whether this observation resulted from direct interaction of cells with RV, or through factors released by cells in response to RV infection. To explore this further, supernatant was harvested from EMT6 and 4T1 murine cell lines after their exposure to RV, DV or no virus and subjected to UV irradiation for five minutes to inactivate RV. An optimization assay was conducted to determine the minimal dose of irradiation time to effectively inactivate RV, with the least disturbance to proteins secreted by cells in the supernatant (Data not shown). When EMT6 cells were exposed to UV-inactivated 4T1 supernatant, there was a significant (*p* < 0.001) increase of PD-L1 expression with supernatant from 4T1 cells treated with RV compared with those treated with DV or no virus (Figure 2E). Similarly, 4T1 cells had a significant (*p* < 0.001) increase of PD-L1 expression with UV-inactivated supernatant from EMT6 cells treated with RV as compared to controls (Figure 2F). Taken together, these data suggest that upregulation of PD-L1 in these cells as a result of RV infection is not due to the virus itself, but rather due to a secreted factor secondary to viral infection.

### 2.3. Reovirus Demonstrates In Vivo Therapeutic Efficacy as a Monotherapy and Is Significantly Enhanced by Combination with PD-1 Inhibition

The EMT6 murine model of BrCa is an established immunocompetent syngeneic model for studying novel immunotherapeutic against this disease. We utilized this model to investigate whether or not RV has therapeutic efficacy against BrCa in vivo and determine the ability of PD-1 inhibition to augment this activity. 

EMT6 tumor-bearing Balb/C mice were treated with monotherapy RV (i.t.), anti-PD-1 antibody (i.p.) or a combination of these agents. Phosphate buffered saline (PBS) (i.p.) was administered as a control. RV treatment was initiated once tumors were palpable (approximately day 6), and repeated on day 9, 12 and 14. Anti-PD-1 antibody was administered on day 14, 17, 20, 23, 26 and 29. In animals treated with both RV and PD-1 blockade, PD-1 blockade was initiated at the conclusion of the RV treatment period, as we hypothesized this would be the point of highest activation of the PD-1/PD-L1 immunosuppressive axis.

**Figure 3:**
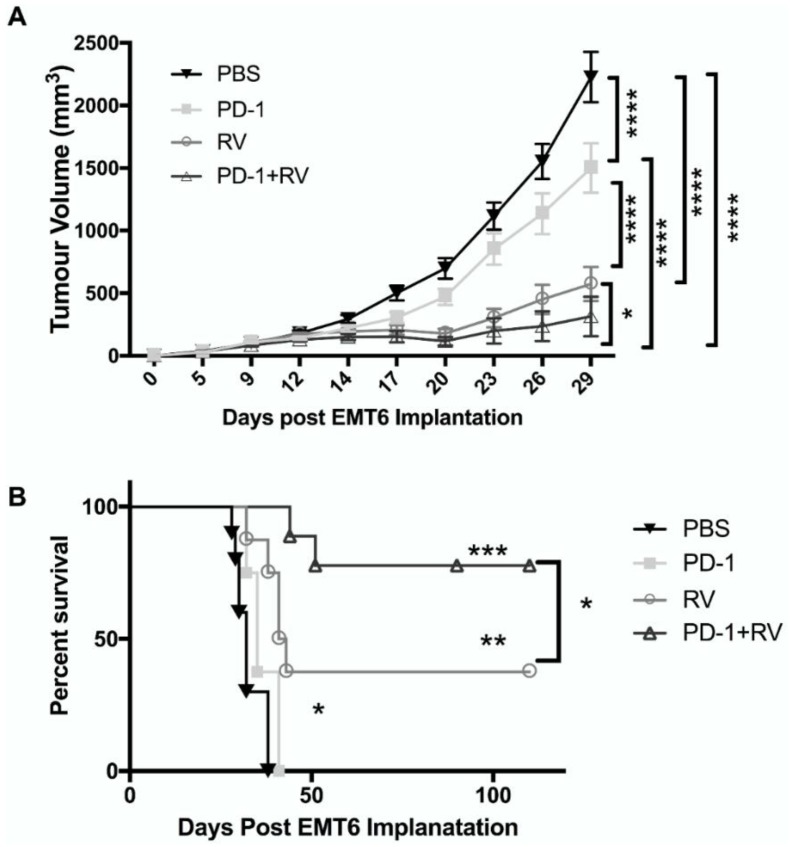
Reovirus combined with PD-1 inhibition results in decreased tumor burden and improved overall survival in the EMT6 murine model. Balb/C mice were implanted with EMT6 (2 × 10^5^ cells) into the right mammary fat pad and treated with phosphate buffered saline (PBS), anti-PD-1 antibody (200 ug i.p.), RV (5 × 10^8^ PFU i.t.) or a combination of these agents. RV was administered four times (days 6, 9, 12 and 14) following tumor implantation and anti-PD-1 antibody was given six times (days 14. 17, 20, 23, 26 and 29). (**A**) Tumor size was followed with caliper measurements every three days starting from day 9. PBS *N* = 15, RV *N* = 13, PD-1 *N* = 13, RV + PD-1 *N* = 14. **** *p* ≤ 0.0001, *** *p* ≤ 0.001, ** *p* ≤ 0.01 and * *p* ≤ 0.05 by two-way. Error bars = SEM of replicates within each group. (**B**) Kaplan–Meier survival plot of mice in each treatment group. PBS *N* = 10, RV *N* = 8, PD-1 *N* = 8, RV + PD-1 *N* = 9. *** *p* ≤ 0.001, ** *p* ≤ 0.01 and * *p* ≤ 0.05 by log rank test. Error bars = SEM of replicates within each group.

Compared to PBS control and anti-PD-1 monotherapy, RV monotherapy significantly reduced tumor burden (Figure 3A). However, the combination of RV and anti-PD-1 significantly enhanced this reduction compared to RV monotherapy (*p* < 0.05) or anti-PD-1 monotherapy (*p* < 0.01). The number of animals used in survival analysis were as follows: PBS (*n* = 10), RV (*n* = 8), anti-PD-1 (*n* = 8) and RV + anti-PD-1 (*n* = 9). Kaplan-Meier analysis revealed an overall survival benefit for mice receiving RV, which was significantly (*p* < 0.05) enhanced by the addition of anti-PD-1 therapy (Figure 3B). In fact, the combination therapy cured ~70% of treated mice (at day 110 post tumor injection). Animals were euthanized when tumors progressed to a size greater than 2 cm in a single dimension, or had weight loss of greater than 20% of body weight, as per institutional Animal Care guidelines. There was no evidence of distant metastases at necropsy, as would be expected with this model [35].

### 2.4. PD-1 Inhibition Augments Reovirus-Mediated Antitumor Immune Response through Recruitment of Memory T-Cell Populations and Enhanced Inflammatory Cytokine Production

**Figure 4:**
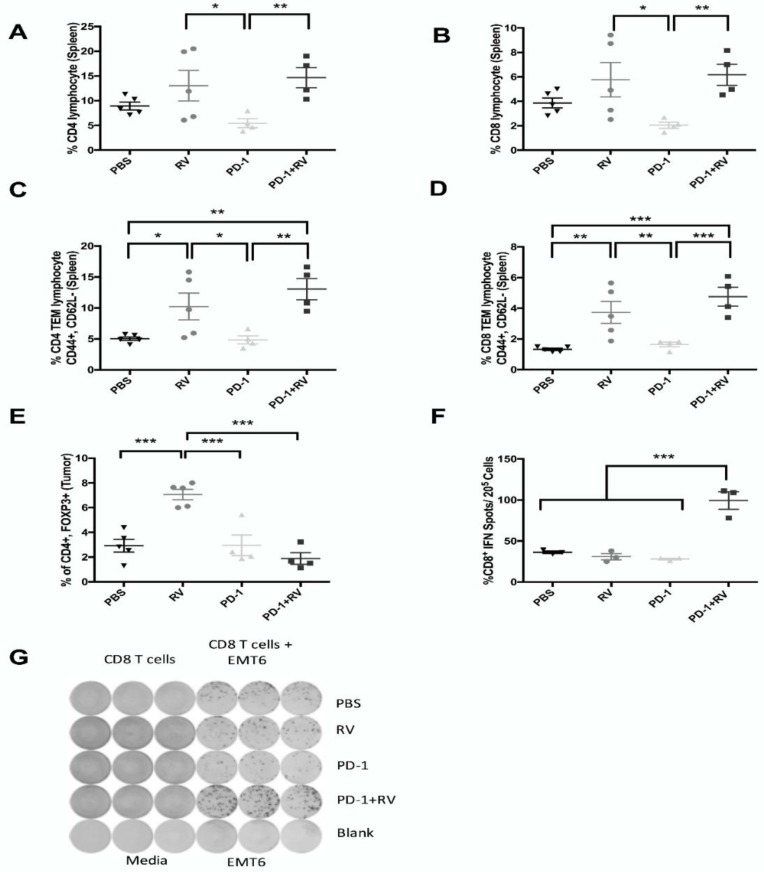
Reovirus combined with PD-1 inhibition enhances splenic immune stimulatory cells while preventing accumulation of tumor immune suppressor cells. Pooled splenocytes (**A**–**D**) and tumor single-cell suspensions (**E**) from EMT6 tumor–bearing mice treated as per Figure 2A were immunophenotyped by flow cytometry. (**A**) CD4^+^ T cells, (**B**) CD8^+^ T cells, (**C**) Effector CD4^+^ memory T cells, (**D**) Effector CD8^+^ memory T cells, (**E**) T-regulatory cells. *N* = 5 mice per group. *** *p* ≤ 0.001, ** *p* ≤ 0.01 and * *p* ≤ 0.05 by one-way ANOVA. Error bars = SEM of experimental replicates. Source of cells indicated in parentheses. CD8^+^ cells were separated from pooled spleens of EMT6 tumor-bearing mice treated as per Figure 2A and stimulated with EMT6 cells. (**F**) Percentage of EMT6-specific IFN^+^ cells determined by ELISPOT assay and (**G**) representative quantification. *N* = 3 mice per group. *** *p* ≤ 0.001 by one-way ANOVA. Error bars = SEM of experimental replicates.

To assess immune subpopulations responsible for the effects of combination treatment, we immunophenotyped splenocytes and tumor-infiltrating immune populations from mice treated as in Figure 3. As such, we found that combination treatment with PD-1 blockade and RV led to a significant increase in splenic CD4^+^ and CD8^+^ T cells, as well as in effector memory populations (CD62L^−^) compared to controls (Figure 4A–D). Furthermore, RV monotherapy led to a significant accumulation of intra-tumoral immunosuppressive regulatory T cells (Tregs). However, the addition of PD-1 blockade significantly reversed this RV-induced effect (Figure 4E). Additionally, combination treatment with RV and PD-1 blockade led to a significant (*p* < 0.001) increase in IFN-γ production by tumor-specific CD8^+^ splenocytes, as determined by the Enzyme-Linked ImmunoSpot (ELISPOT) assay (Figure 4F,G).

**Figure 5:**
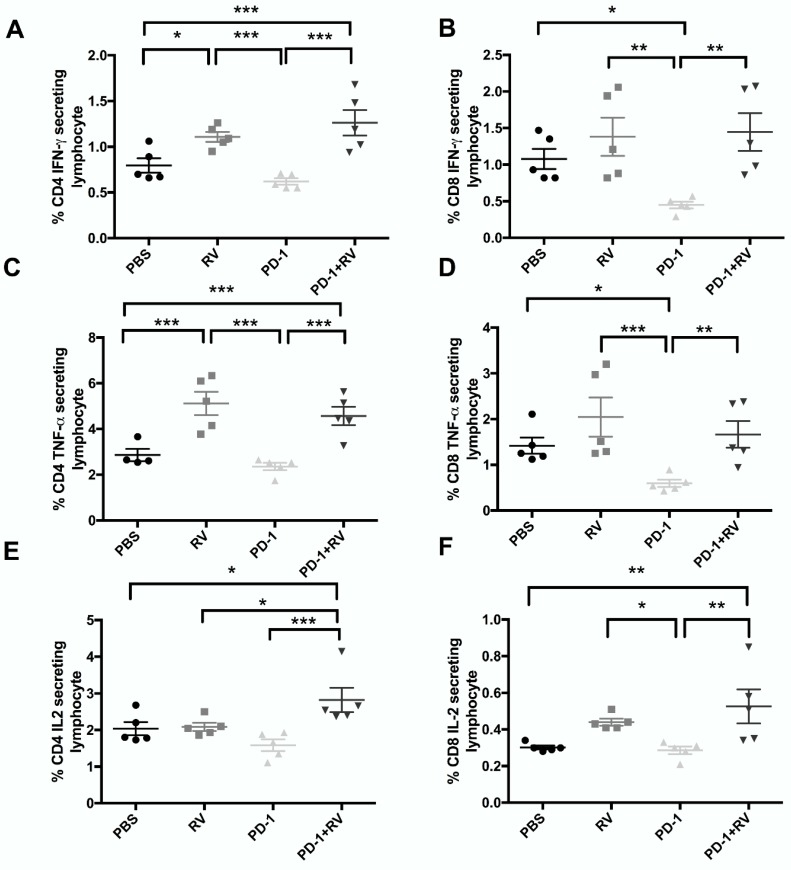
Reovirus combined with PD-1 inhibition significantly enhances IFN-γ, TNF-α and IL-2 production by CD4 and CD8 T cells. Splenocytes were stimulated with Ionomycin for 12 h with Brefeldin A added in the last two hours. Surface and intracellular flow cytometric analysis were performed and stained for markers specific for CD4 T cells (**A**,**C**,**E**) and CD8 T cells (**B**,**D**,**F**). Cytokine secretion for each population was then analyzed [IFN-γ (A/B), TNF-α (C/D) and IL-2 (E/F)]. *N* = 5 per group. *** *p* ≤ 0.001, ** *p* ≤ 0.01 and * *p* ≤ 0.05 by one-way ANOVA. Error bars = SEM of experimental replicates.

Further, to assess changes in immune subpopulations as a result of RV+ PD-1 blockade, we also sought to characterize their capacity to produce inflammatory cytokines. By utilizing intracellular cytokine staining, and flow cytometry, we found that dual treatment significantly enhanced CD4^+^ and CD8^+^ T-cell production of IFN-γ, TNF-α and IL-2 compared to control (Figure 5A–F). Taken together, these data highlight the systemic adaptive antitumor immune response against BrCa that is induced by treatment with RV and PD-1 blockade.

### 2.5. Treatment with Reovirus and PD-1 Induces Protective Immunity against Tumor Re-Challenge, and Treatment Benefits Depend on the Presence of CD8^+^ T Cells

**Figure 6:**
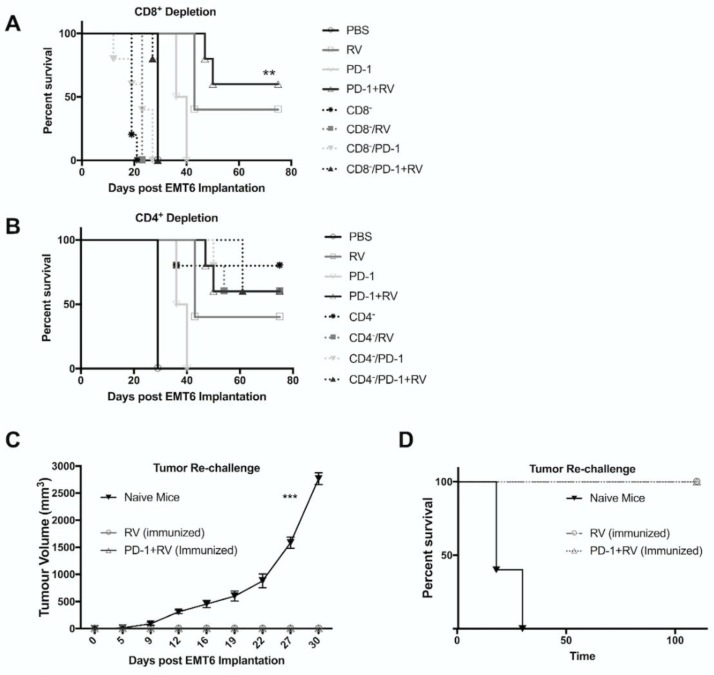
Survival advantage of combination therapy relies on presence of CD8^+^ T cells. (**A**,**B**) Kaplan–Meier plot demonstrating overall survival (OS )for mice pretreated with depleting CD8a (**A**) or CD4a (**B**) antibodies (i.p.) followed by treatment as in Figure 3A,B. ** *p* ≤ 0.01 and * *p* ≤ 0.05 by log-rank test. *N* = 5 mice. Results from Figure 3B included in panel **A**,**B** as reference. (**C**,**D**) Cohorts of pretreated mice demonstrating cure (Combination: *N* = 6, RV: *N* = 3) and a cohort of treatment-naïve mice (*N* = 5) were challenged with EMT6 (1 × 10^5^ cells) into the opposite (left) mammary fat pad as initial tumor inoculation. (**C**) Tumor size was followed with caliper measurements every three days starting from day 9. (**D**) Kaplan–Meier survival plot of mice in each treatment group. ***p ≤ 0.001. Error Bars = SEM of experimental replicates.

In order to further characterize the immunotherapeutic mechanisms underlying the efficacy of RV and combination therapy, T-cell depletion studies were conducted and successful depletion of >98% was confirmed, as per our previously published data [13]. The survival benefit with combination RV and anti-PD-1 therapy was mediated via CD8^+^ T cells (Figure 6A). Interestingly, we noted that CD4 depletion as monotherapy led to a non-significant trend towards therapeutic benefit (Figure 6B). This can possibly be explained by depletion of immunosuppressive regulatory T cells and is consistent with previous findings from our group in a model of renal cell carcinoma, and other reports [13,36]. Notably, CD4^+^ depletion did not decrease the therapeutic benefit of RV, or of combination therapy with anti-PD-1. 

Understanding the importance of the anti-tumor immune response underlying the therapeutic benefit of RV or combination therapy, we sought to assess the capacity of surviving mice to respond against tumor re-challenge. We found that mice previously treated with RV or combination therapy were protected from tumor re-challenge, whereas treatment-naïve mice developed rapidly growing tumors and had decreased survival (Figure 6C,D).These results highlight the establishment of protective immunity following combination therapy with RV and anti-PD-1 antibody.

## 3. Discussion

Immune checkpoint blockade is a therapeutic approach that has recently seen clinical success in a number of tumor types, including lung cancer [37], melanoma [19], and renal cell carcinoma [21]. However, the successful application of these agents for the treatment of BrCa has been lacking, given that most BrCa subtypes have low levels of T-cell infiltration and are thus felt to be immunologically “cold” [38]. Consequently, response rates to single-agent immune checkpoint blockade in patients with BrCa to date have been modest [39,40]. Utilizing oncolytic RV to enhance the immunogenicity of the BrCa tumor environment, in combination with immune checkpoint blockade, is thus an attractive treatment strategy. Further, with RV + paclitaxel demonstrating significant overall survival benefit compared to paclitaxel alone in a phase II trial completed by the Canadian Cancer Trials Group, the utilization of a combined immunotherapeutic strategy is topical. Here we report the first preclinical evidence of the adaptive anti-tumor response against BrCa driven by RV and augmented by PD-1 blockade. This establishes RV + PD-1 blockade as a promising combinatorial approach for the treatment of localized BrCa, and further supports the notion that oncolytic virotherapy can be rationally combined with other immune-modulating treatment strategies.

The conducted in vitro studies highlight the ability of RV to successfully infect and induce oncolysis in a panel of human and murine BrCa cell lines (Figure 1A). Consistent with a previous study [11], T-47D and SKBR-3 were the most sensitive to RV oncolysis of human cell lines assayed, whilst Hs 578T was the most resistant. Importantly, JAM-A expression—which has shown to be crucial in effective RV oncolysis of multiple myeloma cells [9]—has also been implicated as being a poor prognosticator of survival in BrCa [41]. It has previously been demonstrated that T47-D has a high basal expression of JAM-A, whilst Hs578T has very little basal expression of JAM-A [41,42]. Taken together with our RV sensitivity data, this would provide a potentially important role for JAM-A in RV cell-entry in BrCa, and therefore warrants further investigation in future studies. It has also been reported that murine cell lines have higher susceptibility to RV-mediated oncolysis than human cell lines of the same tumor histology [13,43], but in the present study we did not find them to be demonstrably different (Figure 1A).

Further, we demonstrated that RV infection of EMT6 cells in vitro leads to an inflammatory cytokine response, similar to that seen in renal cell carcinoma [13], melanoma [44], and prostate cancer [32]. IP-10 and RANTES are two of the notable chemokines secreted by RV-infected EMT6 cells. Since these molecules are potent chemoattractants of Th1 (anti-viral) lymphocytes, it is possible that they may be involved in effector cell recruitment as seen in our Transwell migration experiments (Figure 1E). Taken together, the data from our in vitro experiments led us to hypothesize that RV could lead to direct oncolysis, and enable an innate and adaptive immune response in vivo.

We also assessed the ability of RV to upregulate BrCa cell expression of the immune checkpoint protein PD-L1. We demonstrated that RV significantly enhanced PD-L1 expression on all human and murine BrCa cell lines tested (Figure 2A–D). Furthermore, Figure 2D,E highlight that the ability for RV to induce PD-L1 expression is not a result of direct viral infection, but rather, is likely secondary to secreted cytokines. IFN-γ is the main cytokine known to induce expression of PD-L1 [45], but interestingly, IFN-γ levels were undetectable in our assessment of inflammatory cytokines. This suggests that other cytokines(s) may be responsible for the noted upregulation in BrCa PD-L1 expression, which will be investigated in future lines of experimentation. Taken together, our data suggested that since RV induces PD-L1 expression in vitro, it might do the same in vivo, thus enhancing the potential for targeting this immunosuppressive axis with PD-1/PD-L1 blockade.

The ability to utilize OV as part of a broader immunotherapeutic strategy will likely be necessary for transition into the clinical arena. In addition to their direct oncolytic effects on BrCa observed in vitro, we hypothesized that their ability to induce a potent anti-tumor immune response could be further augmented by the addition of PD-1 blockade. We observed that this combinatorial treatment strategy led to significant improvement in both tumor burden and overall survival compared to either agent alone in the immunocompetent EMT6 murine model of BrCa (Figure 3A,B). A durable cure was seen up to 110 days of follow-up in ~70% of the mice treated with both RV and PD-1 blockade. Additionally, the presence of tumor-specific CD8^+^ splenic lymphocytes was significantly higher in dually treated animals compared to the other treatment groups, indicating the ability of this treatment approach to generate a systemic adaptive anti-tumor response (Figure 4F). The importance of the adaptive anti-tumor immune response generated from combination treatment can be highlighted with tumor immunity secondary to tumor re-challenge (Figure 6D). Additionally, we found that treatment efficacy was lost as a result of CD8^+^ depletion (Figure 6A), consistent with results from our group [13], and others [46]. Interestingly, there appeared to be a survival advantage imparted to animals when treated with CD4^+^ depletion, which can possibly be explained by the removal of the immunosuppressive Treg population. Furthermore, it is important to note that ~40% of mice treated with RV achieved cure at 110 days, and these mice were also immune to tumor-re-challenge, thus supporting our hypothesis that the addition of PD-1 blockade augments anti-tumor immunity through de-repression of the Th1 IFN-γ T-cell response generated by RV treatment.

As we hypothesized that beneficial treatment effects were due to the establishment of an adaptive anti-tumor response, we assayed immune subpopulations as well as their propensity to produce inflammatory cytokines by flow cytometry. We have shown that treatment with RV enhances the presence of splenic CD4^+^ and CD8^+^ effector memory cells, but the addition of an anti-PD-1 antibody did not augment this effect further (Figure 4C,D). Furthermore, our data show that treatment with RV leads to a significant increase in intratumoral Tregs, which is in keeping with previous observations [13,43,47]. However, the addition of PD-1 blockade reduces Treg numbers equivalent to control. This could underscore the additional survival benefit imparted by combination therapy over RV alone, as in addition to being commonly understood as an immunosuppressive subpopulation, Treg numbers have been a predictor of poor survival in human studies of BrCa [48]. Further, it has recently been suggested that PD-1 blockade abrogates Treg activity, but the mechanism underpinning this relationship is currently unclear [26]. Finally, our data demonstrate that RV treatment significantly increases cytokine (IFN-γ, TNF-α, IL-2) production by CD4^+^ and CD8^+^ T cells, but with the exception of IL-2 production by CD4^+^ lymphocytes, PD-1 blockade does not significantly enhance these. There is some evidence from in vitro studies that Tregs can reduce CD8^+^ lymphocyte production of IL-2 [49], and as such, since dually treated animals had significantly fewer Tregs compared to RV monotherapy it is conceivable that they would have a resultant increase in IL-2 production by effector lymphocytes. However, this theory is speculative in nature. It is also possible that since we only assayed a limited subset of cytokines, there are demonstrable differences in other inflammatory cytokines not tested. Finally, since we looked at cytokine production by CD4^+^/CD8^+^ lymphocytes only, cytokine production by other immune subsets is not accounted for.

Although the results of the present study present promising clinical applicability, there are areas for future exploration. For example, we have demonstrated the production of a potent anti-tumor immune response secondary to RV and anti-PD-1 treatment, but it remains to be seen how intratumoral RV treatment would effect sites of distant disease (abscopal effect). In a murine model of fibrosarcoma, it has been recently shown that oncolytic virotherapy paired with immune checkpoint blockade can reduce tumor burden in both local, and distant lesions [46]. Furthermore, it is not yet clear what the optimal timing of dosing is for both virus and immune checkpoint blockade. We reasoned that initiating anti-PD-1 antibody after RV therapy would allow adequate time for tumor cell lysis, antigen presentation, T-cell priming, and subsequent immune checkpoint upregulation. Furthermore, we rationalized that this protocol timing would limit the anti-viral response during the time RV was being injected intratumorally, as PD-1 blockade has shown to potentiate anti-RV Th1 responses [26]. A recent study by Fend and colleagues [46] corroborates this rationale, as PD-1 blockade demonstrated better efficacy when delivered after seven days of OV therapy, as opposed to before.

Finally, as our study focused on localized BrCa, it will be prudent to assess this synergistic immunotherapeutic strategy in the metastatic setting, in which the immunocompetent metastatic syngeneic 4T1 model could be utilized [50]. Furthermore, a pre-clinical study by Bourgeois-Daigneault et al. [51] has demonstrated the potential for the neo-adjuvant utilization of OV in order to prime an anti-tumor response and engender susceptibility to PD-1 axis blockade in BrCa. As such, given the results of the present study, and the clinical applicability of RV to BrCa, an early-phase clinical trial of neoadjuvant RV is justified.

## 4. Materials and Methods

### 4.1. Cell Lines and Reagents

Breast cancer cell lines (T-47D, SK-BR-3, MDA-MB-468, MCF-7, Hs 578T, EMT6 and 4T1) were obtained from American Type Culture Collection (ATCC). Cell lines were authenticated by ATCC by short tandem repeat analysis prior to purchase. No further authentication was done by the authors. For all experiments, early passage cells were utilized. Cells were grown in Dulbecco’s Modified Eagle’s Medium (DMEM) (Thermo Fisher Scientific, Waltham, MA, USA) supplemented with 1 mM sodium pyruvate and 10% heat inactivated fetal bovine serum (FBS) (Thermo Fisher Scientific, Waltham, MA, USA). EMT6 was maintained in Waymouth’s medium (Thermo Fisher Scientific, Waltham, MA, USA) supplemented with 15% heat-inactivated FBS and 4T1 was maintained in Roswell Park Memorial Institute (RPMI) (Thermo Fisher Scientific, Waltham, MA, USA) medium supplemented with 10% heat-inactivated FBS. Cultures were free of antibiotics and negative for mycoplasma as determined by routine testing. Reovirus (RV) serotype 3 (original stocks received from Oncolytics Biotech, Calgary, AB, Canada) was grown in L-929 cells then purified and titered as previously described [52].

### 4.2. Cell Viability Assay and Determination of RV ED50 

Cells were seeded at a density of 7 × 10^3^ cells/well into 96-well microtitre plates and incubated for 24 h in the appropriate media. Serial dilution of multiplicity of infection (MOI) of RV was then added to each well for 48 h. Following this, WST-1 (Sigma Aldrich, St. Louis, MO, USA) (diluted 10:1) was added to each well and absorbance was quantified utilizing a BioTek Power Wave XS plate reader (Hercules, CA, USA). Percent viability was calculated as the absorbance ratio of treated/untreated cells multiplied by 100. The median effective dose (ED_50_) was determined by calculating the intercept of the log effect vs. log RV dose log. Pictures of RV cytopathic effects at 48 h post-infection were captured with a Zeiss^®^ (Jena, Thuringia, Germany) Axiovert 200M microscope at 10× magnification.

### 4.3. Transwell Chemotaxis Assay

Chemotaxis of DCs and splenocytes were assessed using a Transwell system. Five hundred µL of supernatant from EMT6 +/− RV was added to the lower chamber of a Transwell plate (Corning, New York, NY, USA) with 5-µm pores. 1 × 10^5^ DCs or 1 × 10^6^ splenocytes in 100 µL medium were added to the upper chamber, and plates were incubated for 3 h at 37 °C. Migrated cells in the lower chamber were harvested and counted by hemocytometer.

### 4.4. Cytokine Expression Assay 

Supernatants were harvested from EMT6 cells infected with live or UV-inactivated RV (DV) for 24 h in 6-well plates. The multiplexing analysis was performed using the Luminex™ 100 system (Luminex, Austin, TX, USA) by Eve Technologies Corp. (Calgary, AB, Canada). Thirty-two markers were simultaneously measured in the supernatant using a MILLIPLEX Mouse Cytokine/Chemokine 32-plex kit (Millipore, St. Louis, MO, USA) according to the manufacturer’s protocol. Assay sensitivities for these analytes ranged from 0.2 to 63.6 pg/mL.

### 4.5. Assessment of PD-L1 Expression via Flow Cytometry

Human BrCa cell lines (MDA-MB 468 and Hs 578T) and murine BrCa cell lines (4T1 and EMT6) were plated in their respective serum-free growth media at 5 × 10^5^ cells/well in 6-well plates. Human cell lines were treated with UV-inactivated RV (DV) at ED_50_ +/− 6 ng/mL human rIFN-γ (R&D Systems, Minneapolis, MN, USA) or RV at ED_50_ +/− rIFN-γ for 24 h at 37 °C. Murine cell lines were treated as grouped above, but with murine 3 ng/mL rIFN-γ (R&D Systems, Minneapolis, MN, USA). Cells were then harvested for flow cytometry analysis and supernatants stored at −80 °C for future analysis. Cold fluorescence-activated cell sorting (FACS) buffer composed of PBS with 0.2% FBS and 0.02% NaN_3_ (Thermo Fisher Scientific, Waltham, MA, USA) was utilized to wash harvested cells twice. 4T1 and EMT6 cells were subsequently stained with 0.25 μL of 0.2 mg/mL APC tagged anti-mouse PDL-1 monoclonal antibody or APC tagged IgG2b κ isotype antibody (Clone 10F.9G2 and Clone RTK4530, BioLegend, San Diego, CA, USA) for 30 min at 4 °C in the dark in 50 μL FACS buffer. MDA MB 468 and Hs 578T were stained with 10 μL FITC tagged anti-human PDL-1 monoclonal antibody or FITC tagged IgG_1_ κ isotype antibody (Clone M1H1 and Clone MOPC-21, BD Bioscience, San Jose, CA, USA) before being processed as above. All cells were subsequently rinsed with cold FACS buffer, fixed in 300 μL 1% formalin, subject to flow cytometry analysis using AccuriTM C6 Flow Cytometer, and results were analyzed with BD AccuriTM C6 software (BD, Franklin Lakes, NJ, USA). See Supplementary Material Figure S1A for a representative gating strategy.

### 4.6. 4T1 and EMT6 Supernatant Cross-Incubation and PDL-1 Expression Analysis 

4T1 or EMT6 cells were cultured in 6-well plates at 4 × 10^5^ cells/well for 24 h at 37 °C. A volume of 1 mL UV irradiated supernatants from EMT6 cells with no treatment, DV treatment, or RV treatment was added to 1 mL of RPMI for a complete volume of 2 mL, and replaced previous media for 4T1 cells in each well. Likewise, 1 mL UV-irradiated supernatants from 4T1 cells with the same treatments was added to 1 mL of Waymouth’s medium and replaced previous media for EMT6 cells. Cells were incubated at 37 °C for 24 h, and harvested for PDL-1 expression analysis by flow cytometry using the same procedure as above.

### 4.7. Immunocompetent Syngeneic Murine Breast Cancer Model

All mice in these studies were housed in pathogen-free conditions with food and water ad libitum and treated within procedural guidelines that were approved by the University of Calgary Animal Care Committee (Study ID AC12-0126). Four groups of 12-week-old BALB/c mice (Charles River, Wilmington, MA, USA) were implanted on day 0 with 2 × 10^5^ EMT6 cells subcutaneously (s.c.) into the right mammary pad (*N* = 15/group). Once tumors were palpable (approximately day 6), therapy was initiated. Mice were grouped into cohorts and treated with intraperitoneal (i.p.) PBS, intratumoral live RV (5 × 10^8^ PFU), anti-PD-1 antibody (RMP1-14, BioXcell, Branford, CT, USA) (0.2 mg i.p.), or a combination of these agents (See Figure S2). Specific treatment schedules are outlined in the figure legends. Dosing of these agents was similar to previously conducted studies [13,26,53].

Bi-weekly caliper measurements were taken to monitor tumor burden. Tumor volume was calculated using the formula, volume = 0.52 × (width)^2^ × length. For the tumor challenge experiment, 1 × 10^5^ EMT6 cells were implanted s.c. into the left (opposite) mammary pad BALB/c mice and tumor burden was assessed by caliper measurement. Treatment-naïve Balb/C mice were challenged in the same manner to serve as a control. 

For immune depletion studies, CD8^+^ or CD4 lymphocyte depletion was accomplished by i.p. injection of 0.25 mg depleting anti-CD4a/CD8a mAb (BioXcell; CD8 Clone: 2.43; CD4 Clone GK1.5) on day 6 post tumor implantation, followed by 0.1 mg on day 8, 12 and 19. *N* = 5 mice/group. 

### 4.8. CD8^+^ Enrichment

Spleens were processed as previously described [13] from Balb/C mice bearing EMT6 tumors and enriched for CD8^+^ T cells using an EasySep Mouse CD8^+^ Selection Kit and EasySep magnet as per manufacturer’s protocol (Stem Cell Technologies, Vancouver, BC, Canada).

### 4.9. Immunostaining and Flow Cytometry of In Vivo Samples

Flow cytometry was performed as previously described [54]. Cell staining was performed using FITC, Alexa Flour 488, PE, APC, PE-Cy7 or PerCP Cy5.5 conjugated rat mAbs against CD4, CD8, CD25, CD44, Foxp3, CD62L, IL-2 (BD Bioscience, San Diego, CA, USA), IFN-γ, and TNF-α (eBioscience, San Diego, CA, USA). Intracellular cytokine staining was done using the Fixation and Permeabilization Solution Kit (BD Bioscience, San Diego, CA, USA) according to the manufacturer’s protocol. This included stimulation of splenocytes with Ionomycin for 12 h, with Brefeldin A being added for the final two hours. Intracellular Foxp3 staining was done using Mouse Foxp3 Buffer Set (BD Bioscience, San Jose, CA, USA) according to the manufacturer’s protocol. Intratumoral lymphocytes were stained and analyzed by flow cytometry following collagenase type I (Gibco, Waltham, MA, USA cat #17100-017) treatment according to the manufacturer’s protocol. Briefly, tumor tissue was washed with PBS, cut into small pieces (<1 × 1 mm), and incubated with collagenase type I (100 U/μL) for 4 h at 37 °C. Tumor fragments were then disaggregated through 100-μm cell strainers, and single cells were collected and enumerated. Samples were run using the BD Accuri flow cytometer and analyzed by FlowJo software (Ashland, OR, USA). See Figures S1B, S3A,B and S4A for representative gating strategies for immune subpopulations.

### 4.10. ELISPOT Assay

ELISPOT assay was conducted as previously described [13]. Briefly, CD8^+^ splenocytes from EMT6 tumor-bearing Balb/C mice were isolated from pooled cell suspensions. 2 × 10^5^ cells were subsequently co-cultured with EMT6 cells for 48 h in 96-well nitrocellulose membrane plates pre-coated with anti-mouse IFN-γ mAb (BD Biosciences, San Diego, CA, USA cat # 552569). As CD8^+^ splenocytes were stimulated by re-introduction to EMT6 cells, they were not pre-stimulated with Ionomycin/PMA. Detection antibody solution was added at 100 mL/well for 2 h followed by Streptavidin-HRP solution at 100 mL/well for a one hour incubation period. AEC substrate was then added and spots were scanned and counted using the CTL-ImmunoSpot S6 Macro Analyzer and BioSpot Software, respectively (CTL, Shaker Heights, OH, USA). The frequency of EMT6-specific CD8^+^ splenocytes was calculated on the basis of the percentage of CD8^+^ T cells present in the responding population.

### 4.11. Statistics

Statistical analysis was performed utilizing GraphPad version 6. Both one- and two-way ANOVA tests were used to determine significance between experimental groups. Kaplan–Meier analysis together with log-rank sum test were utilized to determine significant in vivo survival benefits. Statistical significance was defined as *p*-values being <0.05 unless otherwise stated. 

## 5. Conclusions

Overall, this study demonstrates the utility of oncolytic RV for the treatment of BrCa, and how this immunotherapeutic approach can be augmented with immune checkpoint inhibition. The addition of systemic anti-PD-1 antibody to intratumoral RV therapy significantly increased the presence of tumor-specific CD8^+^ T cells and reduced the number of intra-tumoral Tregs. These mechanisms likely underscore the significant reduction in tumor burden, and significantly improved survival. Furthermore, these therapeutic effects were found to be dependent on the presence of CD8^+^, and not CD4^+^ T Cells. Thus, combination therapy with RV and an anti-PD-1 antibody represents a rationale immunotherapeutic strategy for the treatment of BrCa and warrants further investigation in a clinical setting.

## Supplementary Materials

The following are available online at http://www.mdpi.com/2072-6694/10/6/205/s1, Figure S1: Representative flow cytometry gating strategies. Figure S2: Representative diagram of the in vivo experimental protocol. Figure S3: Representative flow cytometry gating strategies. Figure S4: Representative flow cytometry gating strategies.
