# Supplementary material for: Oncolytic Reovirus and Immune Checkpoint Inhibition as a Novel Immunotherapeutic Strategy for Breast Cancer
Source: Cancers (Basel). 2018 Jun 15;10(6):205. doi: 10.3390/cancers10060205 (PMC6025420; doi:10.3390/cancers10060205)
Supplement: Supplementary file 1 [file cancers-10-00205-s001.pdf]

**** The editor has said, "Please correct the mismatches found between the labeling of supplementary figures and the legends." It is unclear what mismatches there are below… please clarify.

Formatted: Font: Palatino Linotype, 10 pt

Appendix

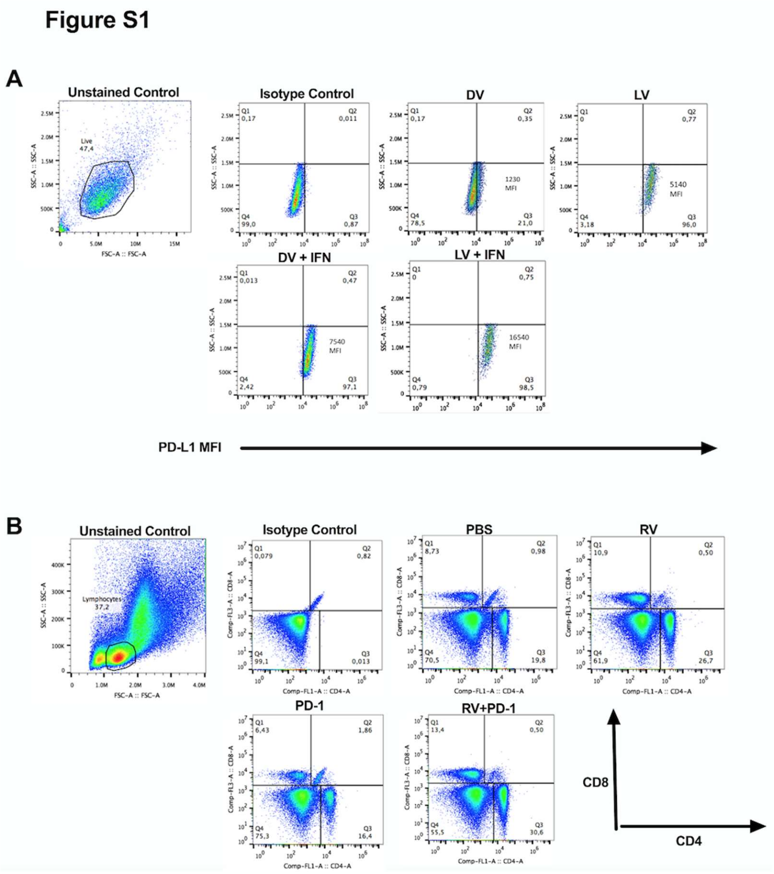

Figure S1. Representative flow cytometry gating strategies

A) PD-L1 expression on BrCa cell lines. Representative plot from MDA MB 468.

B) Quantification of CD4+/CD8+ splenocytes from the *in vivo* EMT6 study. Representative plot shown.

Figure S2

A

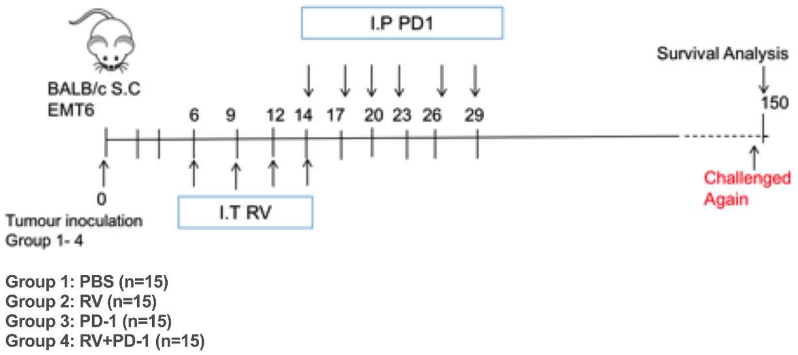

Figure S2. Representative diagram of the *in vivo* experimental protocol

A) Four groups of 12-week old BALB/c mice (Charles River) were implanted on day 0 with 2 x 10^5 EMT6 cells subcutaneously (s.c) into the right mammary pad (N=15/group). Once tumors were palpable (approximately day 6), therapy was initiated. Mice were grouped into cohorts and treated with i.p PBS, intratumoral live RV (5x10^8 PFU), anti-PD-1 antibody (RMP1-14, BioXcell, Connecticut, USA)(0.2 mg i.p.), or a combination of these agents.

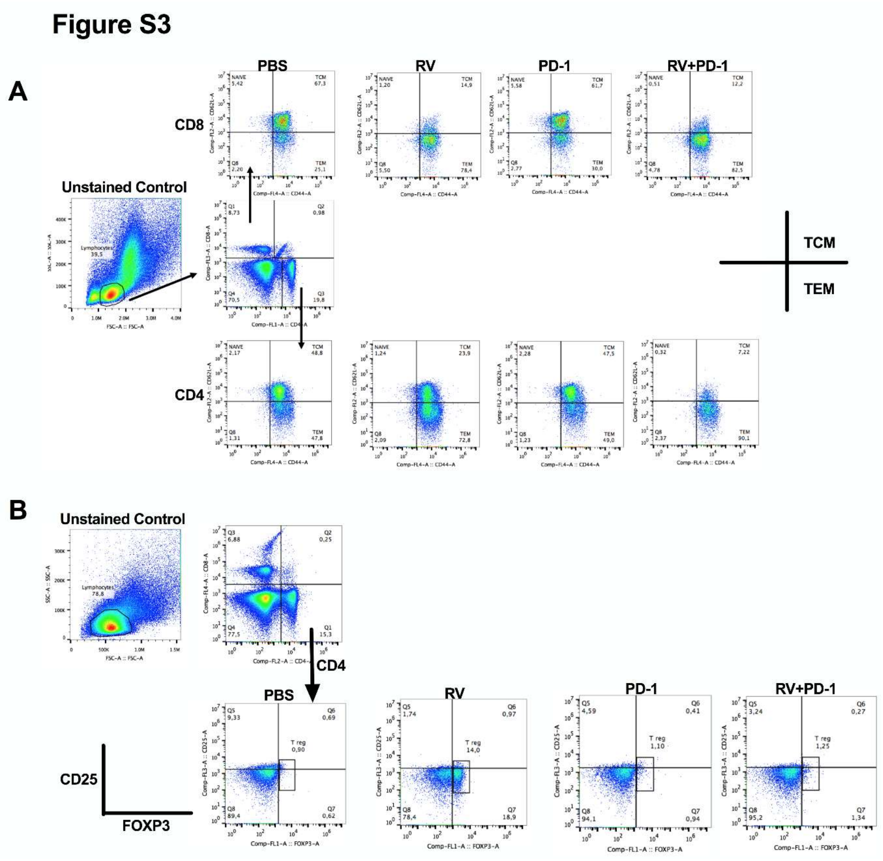

Figure S3. Representative flow cytometry gating strategies

A) Quantification of memory $CD4^+$/$CD8^+$ T cells (TEM, TCM) from the *in vivo* EMT6 study. Representative plot shown.

B) Quantification of Treg ($CD4^+$ $CD25^{lo}$ $FOXP3^+$) from *in vivo* EMT6 study. Representative plot shown.

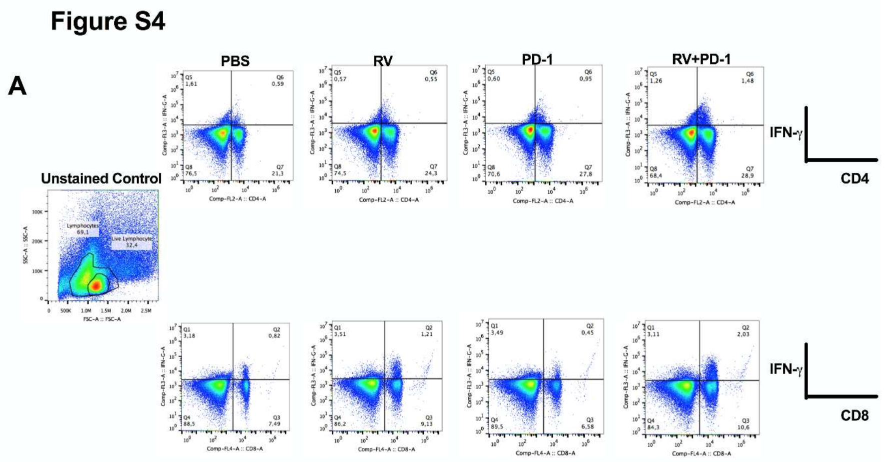

Figure S4. Representative flow cytometry gating strategies

A) Quantification of intracellular cytokine production from CD4+/CD8+ splenocytes from the *in vivo* EMT6 study. Representative plot of IFNγ shown.
